# Early predictors and outcomes of mechanical circulatory support use during single lung transplant for secondary pulmonary hypertension: A large single-center experience

**DOI:** 10.1016/j.jhlto.2026.100587

**Published:** 2026-05-04

**Authors:** Sean M. Baskin, Danielle A. Pulton, Dhruv Vasant, Y. Yuliana Salamanca-Padilla, Hauqing Zhao, Ke Cheng, Hiromu Kehara, Norihisa Shigemura, Sankalp Sehgal

**Affiliations:** a Department of Anesthesiology, Temple University Lewis Katz School of Medicine, 3401 N. Broad Street, Philadelphia, PA; b Center for Biostatistics and Epidemiology, Department of Biomedical Education and Data Science, Temple University School of Medicine, Philadelphia, PA; c Standard Process Inc. 1200 W. Royal Lee Drive, Palmyra, WI 53156; d Department of Surgery, Division of Cardiovascular Surgery, Temple University Lewis Katz School of Medicine, 3401 N. Broad Street, Philadelphia, PA; e Department of Cardiovascular Surgery, University of Pittsburgh School of Medicine, 200 Lothrop Street, Suite C-920, Pittsburgh, PA 15213; f Department of Anesthesiology, Division of Cardiac Anesthesia, Boston University Chobanian and Avedisian School of Medicine and Boston Medical Center, 725 Albany St 7th Floor, Boston, MA 02118

**Keywords:** Lung transplant, Pulmonary hypertension, Mechanical circulatory support, ECMO, RV-PA coupling ratio

## Abstract

**Background:**

Minimal data exists to predict the need for mechanical circulatory support (MCS) for patients with secondary pulmonary hypertension (SPH) undergoing single lung transplant (SLT). We aim to identify preoperative hemodynamic and echocardiographic variables that could predict MCS use and compare clinical outcomes for SLT with and without intraoperative MCS use.

**Methods:**

An institutional registry of SLT recipients with SPH between 2017 and 2021 were analyzed. Cohorts were separated by the use of intra-operative MCS and analyzed. Odds ratios and multivariable logistic regression were utilized to evaluate the likelihood of requiring intraoperative MCS during SLT.

**Results:**

We identified 293 patients, of which 44 (15.1%) required intraoperative MCS use, while 249 (84.9%) did not. Preoperative RV-PA coupling ratio or TAPSE/PASP ratio <0.3 was highly associated with intraoperative MCS use during SLT (OR4.09 (95% 2.06 – 8.14, p=<0.0001). PCWP, pre-operative PVR, transplant laterality, LAS and intraoperative PA Systolic Pressure were also independently associated with intraoperative MCS use. Length of stay was longer in the MCS-use group (29.8 vs 18.5 days, p= 0.007) whereas survival was similar at 30 (100% MCS vs 99.6% non-MCS, p=1) and 60 days (100% MCS vs 98% non-MCS, p=0.75). A Kaplan-Meier analysis showed no survival difference to 3 years (p=0.92).

**Conclusions:**

Assessment of preoperative RV-PA uncoupling using TAPSE/PASP ratio can help predict intraoperative MCS use during SLT. A preoperative TAPSE/PASP <0.3 confers a 4-fold increase in the likelihood of MCS use. The use of MCS during SLT does increase the length of stay, however, does not significantly change survival.

## Introduction

Single Lung Transplantation (SLT) has been shown to be an effective treatment in patients with end-stage lung disease attributable to severe secondary pulmonary hypertension (SPH).^1^ In comparison to the double lung transplantation (DLT) patient population, SLT patients tend to be older and frail with multiple co-morbidities. Due to limited organ availability, SLT offers reduced waitlist time and waitlist mortality, as well as overall low operative mortality, with a 3-year survival reported to be between 55% and 79%^2^, ^3^, ^4^, ^5^

While current guidelines such as the International Society for Heart and Lung Transplantation (ISHLT) consensus statement support mechanical circulatory support (MCS) during lung transplant (LT), most commonly for patients with at least moderate pulmonary hypertension (PH), no specific selection criteria has been firmly established.^1^, ^6^ Intraoperative MCS use during lung transplant provides the benefit of hemodynamic stability and may help reduce rates of primary graft dysfunction, albeit an increase in the risk of complications such as inflammation and bleeding and no clear survival benefit.^7^, ^8^, ^9^ Furthermore, there remain significant variations in clinical practices in part due to institutional policies, surgical preference, and resource availability.^10^

In this study, we aim to 1) identify preoperative hemodynamic and echocardiographic variables that may help predict MCS use in SLT patients and 2) compare clinical outcomes of patient groups undergoing SLT with and without intraoperative MCS. The findings of this study would help evaluate current practices of MCS use during SLT and identify a target population for its selective use.

## Methods

### Patient selection and study variables

After obtaining approval and consent waivers from the Temple University Institutional Review Board, an institutional registry of lung transplant recipients who underwent single lung transplant between 2017 and 2021 was queried. Adult patients greater than 18 years old with at least mild secondary pulmonary hypertension (SPH), defined as mean pulmonary arterial pressure (mPAP) >20 mmHg, were identified.^11^ Exclusion criteria were reported mPAP < 20mmHG, double lung transplant (DLT) procedures, missing data, pre-operative extracorporeal membrane oxygenation (ECMO) support, or intraoperative cardiopulmonary bypass (CPB) to repair a cardiac structural injury as noted in the operative report, or a concomitant cardiac surgery. Patients with idiopathic pulmonary arterial hypertension were also excluded as they are generally treated with DLT.

Hemodynamic and echocardiographic variables were derived from the most recent preoperative right heart catheterization (RHC) and transthoracic echocardiography (TTE) findings prior to the transplantation date, including pulmonary vascular resistance (PVR), central venous pressure (CVP), cardiac index (CI), tricuspid annular plane systolic excursion (TAPSE), Pulmonary Arterial Systolic Pressure (PASP), Pulmonary Arterial Diastolic Pressure (PADP), mPAP and TAPSE/ PASP ratio. Institutional practice is for pre-transplant TTEs to be repeated every 3–6 months and RHC performed at least annually once the patient is listed for transplant. Intraoperative hemodynamics from the pulmonary-artery catheter (PASP, mPAP, PADP) after the induction of anesthesia but prior to surgical incision and the use of MCS were extracted from the anesthesia and surgical records. Right Ventricle-Pulmonary Arterial (RV-PA) Coupling Ratios were calculated using TTE-derived TAPSE and invasive PASP measurements and described as the ratio TAPSE/PASP. Postoperative outcomes including length of stay and survival for up to 3 years were also extracted from the internal registry using data tracking via electronic medical record (EMR). Survival at 3 years was chosen to allow consistent potential survival windows for the entire data set.

### Intraoperative management

Standard institutional intraoperative management for lung transplant was performed for all patients, which included arterial and central venous access with a pulmonary artery catheter (PAC) placement, as well as transesophageal echocardiography (TEE). Lung isolation for left-sided transplants was carried out with a single lumen endotracheal tube and a bronchial blocker while right-sided transplants were performed using a left-sided double lumen endotracheal tube. Per institutional protocol, all patients were administered inhaled nitric oxide for pulmonary vasodilation from the time of intubation and continued post-operatively for at least 12 h. The decision to use MCS was undertaken intra-operatively at the joint discretion of the anesthesiologist and transplant surgeon without specific protocolization. The type of MCS (Veno-Arterial ECMO or CPB) and the timing of its initiation was also at the joint discretion of the anesthesiologist and transplant surgeon. No patient was supported intraoperatively with Veno-Venous ECMO. The transplant surgeon selected the cannulation location, technique, and cannulas. Standard practice is peripheral cannulation via cutdown technique. Central cannulation was performed selectively at the discretion of the transplant surgeon. Given surgeon preference for ECMO vs CPB, both platforms were collectively grouped as MCS-assisted to avoid a per-surgeon analysis.

### Statistical analysis

We compared the demographic variables and comorbidities for patients undergoing single lung transplant with and without MCS. Categorical variables were analyzed using Chi-squared test while continuous variables were analyzed using two sample t-tests between two groups. We used the Kaplan-Meier method to assess survival for over 3 years. Multivariable Logistic regression models were constructed to evaluate the likelihood of requiring intraoperative MCS using variables that met statistical significance on univariate analyses. P=0.05 on 2-sided significance test was considered statistically significant for all analyses. The optimal cut point for the preoperative TAPSE/PASP ratio as a predictor of intraoperative MCS use was determined from the area under the receiver operating characteristic (ROC) curve computed on the continuous TAPSE/PASP ratio, with the threshold selected to maximize the Youden index (*J* = sensitivity + specificity − 1). The data-derived optimum was 0.32; for ease of clinical interpretation and bedside use, this value was rounded to 0.3, which was then applied as the dichotomization threshold in all subsequent analyses.

Statistical analyses were conducted with SAS 9.4 (SAS Institute Inc., Cary, NC).

## Results

### Pre-transplant evaluation

**Figure 1:**
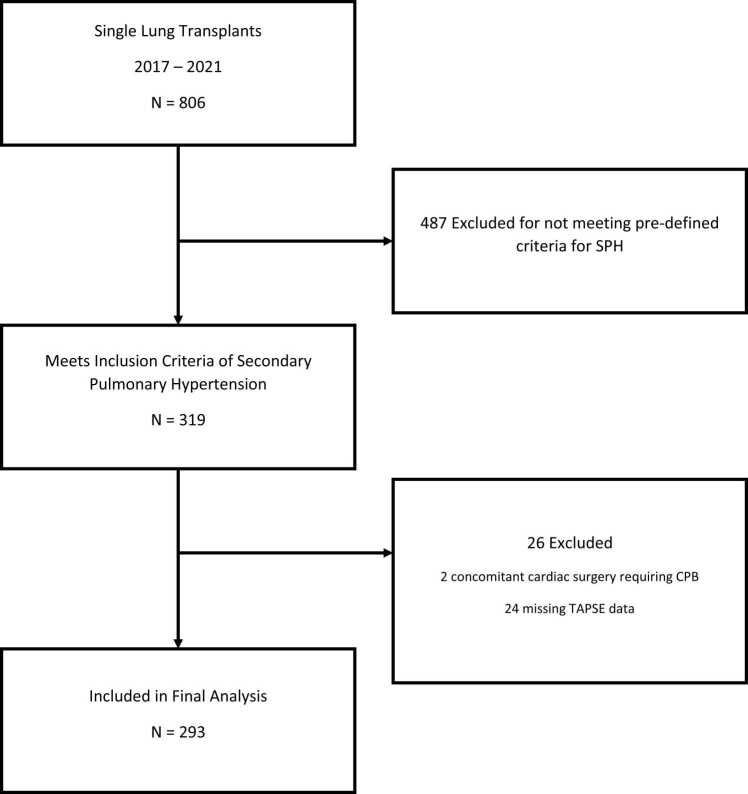
STROBE Diagram.

A total of 806 patients underwent single lung transplant between 2017 and 2021, of which 293 patients were included in the final analysis (Figure 1). Of the 293 included transplants, 249 (85%) were performed without intraoperative MCS use, while 44 (15%) needed intraoperative MCS. Baseline demographics between the two groups are shown in Table 1. There were no statistically significant differences between the groups with respect to demographics such as age, sex, race, height and weight. Preoperative total lung capacity (TLC), left ventricular ejection fraction (LVEF), cardiac output (CO) and cardiac index (CI) did not differ either. Preoperative PVR was significantly higher in the MCS-assisted group (4.6 vs 3.0WU, p<0.0001), as was the Lung Allocation Score (LAS) (55.9 vs 40.1 p=<0.0001) (Table 2). There was no significant difference in pre-transplant TAPSE between MCS-assisted and no-MCS assisted patients (TAPSE 19.1 mm vs 18.2 mm, p=0.18) although when TAPSE was dichotomized into reduced (≤16 mm) or normal (≥17 mm), a reduced TAPSE was found to be associated with an increased use of intraoperative MCS use, however, this did not reach statistical significance (20.9% vs 12.6%, p=0.068). The primary diagnosis requiring transplant also differed between the 2 groups. Restrictive lung disease was the primary indication for transplant in 35 (79.5%) of those who required MCS while only 135 (54.2%) of those patients who did not require MCS (Table 2).

**Table 1:** Baseline Demographics

**Variable**	**Total**	**With Intraoperative MCS**	**Without Intraoperative MCS**	**p-value**
	**(n=293)**	**(n=44)**	**(n=249)**	
Patient Age, mean±SD, yrs	66.2±7.1	66.6±9.0	66.1±6.7	0.7
Sex, N (%)				0.32
Female	107 (36.5)	19 (43.2)	88 (35.3)	
Male	186 (63.5)	25 (56.8)	161 (64.7)	
Race, N (%)				0.7
Black	24 (8.2)	5 (20.8)	19 (79.2)	
Others/Unknown	32 (10.9)	5 (15.6)	27 (84.4)	
White	237 (80.9)	34 (14.3)	203 (85.7)	
BMI, mean±SD, kg/m^2^	27.7±4.5	28.4±4.8	27.6±4.4	0.28
Height, mean±SD, meters	1.7±0.1	1.7±0.1	1.7±0.1	0.4
Weight, mean±SD, kg	77.8±16.1	78.7±17.3	77.6±15.9	0.68
BSA, mean±SD, m^2^	1.9±0.2	1.9±0.3	1.9±0.2	0.89
Preoperative TLC mean±SD, L	5.94±1.08	5.79±1.13	5.96±1.08	0.32
Preoperative EF, mean±SD, %	59.7%±6.3	60.1%±6.5	59.7%±6.3	0.69
Preoperative Cardiac Index, mean±SD, L/min/m^2^	2.5±0.5	2.4±0.6	2.5±0.5	0.1
Preoperative Cardiac Output, mean±SD, L/min	4.7±1.0	4.5±1.2	4.8±0.9	0.11

BMI: body mass index; BSA: Body surface area; EF: Ejection Fraction; RHC: right heart catheterization; SD: standard deviation; TLC: total lung capacity

**Table 2:** Analysis of Preoperative Variables

**Variable**	**Total**	**With Intraoperative MCS**	**Without Intraoperative MCS**	**P-value**	**Odds Ratio (95% CI)**
	**(n=293)**	**(n=44)**	**(n=249)**		
Preoperative CVP mean±SD, mmHg	4.9±3.4	4.2±4.1	5.0±3.2	0.12	0.77 (0.56, 1.06)
Preoperative PCWP, mean±SD, mmHg	9.2±4.9	7.3±4.5	9.5±4.9	0.006	0.71 (0.56, 0.91)
Preoperative PVR, mean±SD, WU	3.3±1.8	4.6±2.9	3.0±1.4	<.0001	1.52 (1.27, 1.81)
Primary Diagnosis Requiring Lung Transplant N (%)				0.003	
COPD	92 (31.4)	2 (4.5)	90 (36.1)		1
CPFE	27 (10.8)	6 (13.6)	21 (8.4)		12.86 (2.42, 68.25)
ILD	21 (7.2)	8 (18.2)	13 (5.2)		27.7 (5.29, 145)
IPF	149 (59.8)	27 (61.4)	122 (48.9)		9.96 (2.31, 42.96)
Other	4 (1.4%)	1 (2.2)	3 (1.2)		15 (1.05, 214.8)
Lung Allocation Score, mean±SD	42.5±14.4	55.9±20.1	40.1±11.6	<.0001	1.33 (1.21, 1.46)
Preoperative TAPSE, mean±SD, mm	19.0±4.4	18.2±5.5	19.1±4.1	0.18	0.86 (0.68, 1.08)
TAPSE Normal/Reduced, N (%)				0.071	
Normal (≥17 mm)	207 (65.5)	26 (59.1)	181 (72.7)		1
Reduced (<16 mm)	86 (27.2)	18 (40.9)	68 (27.3)		1.84 (0.95, 3.57)
TAPSE/PASP <0.3, N (%)	58 (19.8)	19 (43.2)	39 (15.7)	<0.0001	4.09 (2.06, 8.14)

COPD: chronic obstructive pulmonary disease; CPFE: combined pulmonary fibrosis and emphysema; CVP: Central Venous Pressure; ILD: interstitial lung disease; IPF: interstitial pulmonary fibrosis; PCWP: pulmonary capillary wedge pressure; PVR: pulmonary vascular resistance; PASP: Pulmonary Arterial Systolic Pressure; SD: Standard Deviation; TAPSE: Tricuspid Annular Plane Systolic Excursion

**Figure 2:**
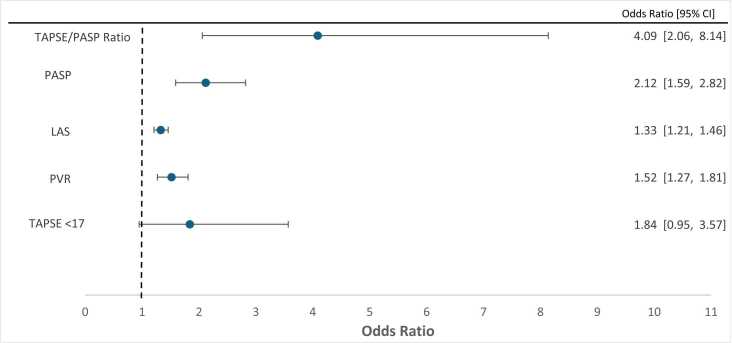
Forest Plot of Odds Ratios of Intraoperative MCS Requirement. LAS: Lung allocation score; PASP: Pulmonary arterial systolic pressure; PVR: Pulmonary vascular resistance; TAPSE: tricuspid annular plane systolic excursion; TAPSE/PASP: Tricuspid Annular Plane Systolic Excursion/ Pulmonary Artery Systolic Pressure.

To identify preoperative variables associated with intraoperative MCS use, patient clinical, hemodynamic, and echocardiographic predictors were evaluated by univariate logistic regression, and the results are summarized as unadjusted odds ratios (ORs) with 95% confidence intervals (CIs) in Figure 2. The continuous preoperative right ventricular–pulmonary arterial coupling ratio (RV-PA), or TAPSE/PASP ratio was first examined by receiver operating characteristic (ROC) analysis, which yielded an area under the curve of 0.70 (95% CI 0.60–0.79). The Youden-optimal cut point was 0.32 (sensitivity 43.2%, specificity 84.3%); for ease of clinical use this value was rounded to 0.3, which preserved the same operating characteristics in the cohort. All subsequent dichotomized analyses therefore used a TAPSE/PASP threshold of 0.3. A preoperative TAPSE/PASP < 0.3 was strongly associated with intraoperative MCS use (unadjusted OR 4.09, 95% CI 2.06–8.14, p < 0.0001; Table 2).

### Intraoperative hemodynamics and post-transplant outcomes

**Figure 3:**
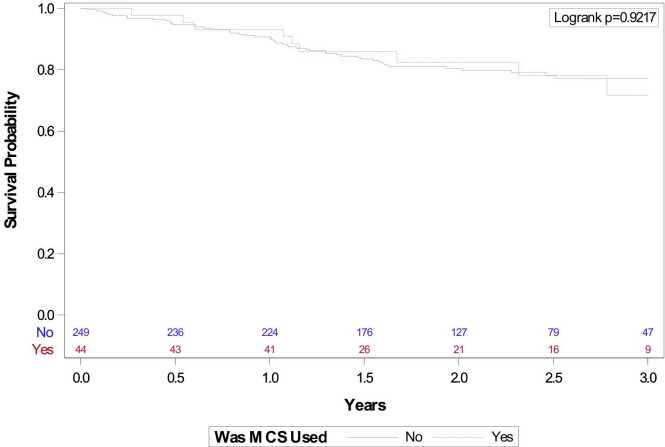
Kaplan Meier Graph of 3-Year Survival After Single Lung Transplant With or Without Intraoperative MCS. MCS: Mechanical circulatory support.

Table 3 reports on intraoperative hemodynamics and post-transplant outcomes. Uniformly, intra-operative PA pressures were significantly higher in the MCS-assisted group. The length of stay was significantly longer in the MCS-assisted group than the non-MCS group (29.8 vs 18.5 days, p= 0.007). Length of mechanical ventilation differed between both groups when categorized into <48 h, ≥48 h – 5 days, and > 5 days with MCS- supported patients requiring longer mechanical ventilation (61.3% vs 80.3%, 18.2% vs 9.2%, 20.5% vs 10.4% respectively, p=0.025). Post-operative hemodialysis, post-operative tracheostomy and grade 3 PGD (or severe PGD) requiring ECMO were similar in both groups. Survival was similar at both 30 days (100% MCS vs 99.6% non-MCS, p=1) and 60 days (100% MCS, 98% non-MCS, p=0.75). Survival at 3 years is shown in a Kaplan Meyer curve (Figure 3, p=0.92) and found no significant difference between the MCS and non-MCS groups (79.6% vs 79.9%, p=0.95).

**Table 3:** Analysis of Intraoperative Hemodynamics and Post-operative Outcomes

	**Total**	**With Intraoperative MCS**	**Without Intraoperative MCS**	**p-value**	**Odds Ratio (95% CI)**
	**(n = 293)**	**(n = 44)**	**(n = 249)**		
Transplant Laterality, N (%)				0.081	
Left	149 (50.9)	17 (38.6)	132 (53.0)		1
Right	144 (49.1)	27 (61.4)	117 (47.0)		1.79 (0.93, 3.45)
Intraoperative PASP, mean±SD, mmHg	46.7±11.5	55.9±14.0	45.1±10.2	<.0001	2.12 (1.59, 2.82)
Intraoperative PADP, mean±SD, mmHg	23.8±7.0	27.7±8.4	23.2±6.5	0.0002	1.54 (1.23, 1.92)
Intraoperative mPAP, mean±SD, mmHg	32.9±8.1	38.9±9.9	31.9±7.4	<.0001	1.60 (1.32, 1.95)
Survival 30 days, N (%)	292 (99.7)	44(100)	248 (99.6)	1	-
Survival 60 days, N (%)	288 (98.3)	44 (100)	244 (98)	0.75	-
Survival 3 years, N (%)	234 (79.9)	35 (79.6)	199 (79.9)	0.95	1.02 (0.46, 2.27)
Length of Stay, mean±SD, Days	20.2±21.0	29.8±35.4	18.5±16.9	0.007	1.09 (1.02, 1.17)
PGD Requiring ECMO, N (%)	14 (4.8)	3 (21.4)	11 (78.6)	0.49	1.58 (0.42, 5.92)
Length of Mechanical Ventilation, N (%)				0.025	2.58 (1.05, 6.33)
< 48 h	31 (10.6)	8 (18.2)	23 (9.2)		**1**
48 h - 5 days	227 (77.5)	27 (61.3)	200 (80.3)		**2.58 (1.05, 6.33)**
> 5 days	35 (11.9)	9 (20.5)	26 (10.5)		**2.56 (1.09, 6.05)**
Post operative Hemodialysis, N(%)	4 (1.4)	1 (2.3)	3 (1.2)	0.58	1.90 (0.19, 18.68)
Post operative Tracheostomy N, (%)	27 (9.2%)	7 (15.9)	20 (8.0)	0.1	2.17 (0.86, 5.48)

ECMO: Extracorporeal Membrane Oxygenation; mPAP: Mean Pulmonary Arterial Pressure; PADP: Pulmonary Arterial Diastolic Pressure; PAPS: Pulmonary Arterial Systolic Pressure; PGD: Primary Graft Dysfunction; SD: Standard Deviation

### Predictors of MCS use during single lung transplant

On univariate analysis, intraoperative MCS use was also associated with higher preoperative PVR, higher LAS, higher intraoperative pulmonary artery systolic pressure (PASP), and a reduced TAPSE < 17 mm (Figure 2). Right-sided transplant was more likely than left-sided to require intraoperative MCS (18.8% vs 11.4%, p = 0.081), but that did not reach statistical significance. Variables that reached p < 0.05 on univariate analysis were carried forward into the multivariate model described below.

**Table 4:** Comparison of Normal or Reduced TAPSE

	**Overall**	**Normal TAPSE (≥17 mm)**	**Reduced TAPSE (≤16 mm)**	**p-value**
	**(n=293)**	**(n=207)**	**(n=86)**	
Preoperative CVP, mean±SD, mmHg	4.9±3.4	4.9±3.3	4.8±3.5	0.84
Preoperative PCWP, mean±SD, mmHg	9.2±4.9	9.1±4.8	9.4±5.1	0.66
Preoperative PVR, mean±SD, WU	3.3±1.8	3.1±1.5	3.8±2.4	0.006
Preoperative Cardiac Index, mean±SD, L/min/m^2^	2.5±0.5	2.5±0.5	2.4±0.5	0.16
Preoperative Cardiac Output, mean±SD, L/min	4.7±1.0	4.8±1.0	4.5±0.9	0.046
Intraoperative PASP, mean±SD, mmHg	46.7±11.5	45.9±11.4	48.7±11.7	0.054
Intraoperative PADP, mean±SD, mmHg	23.8±7.0	23.5±7.0	24.6±6.9	0.23
Intraoperative mPAP, mean±SD, mmHg	33.2±8.1	32.7±8.1	37.3±8.1	0.14
Lung Allocation Score, mean±SD	42.5±14.4	40.8±12.9	46.6±16.9	0.005
Transplant Laterality, N (%)				0.047
L	149 (50.9)	113 (54.6)	36 (41.9)	
R	144 (49.1)	94 (45.4)	50 (58.1)	
Was MCS Used, N (%)				0.068
No	249 (85.0)	181 (87.4)	68 (79.1)	
Yes	44 (15.0)	26 (12.6)	18 (20.9)	
TAPSE/PASP, N (%)				<.0001
<0.3	58 (19.8)	12 (5.8)	46 (53.5)	
>=0.3	235 (80.2)	195 (94.2)	40 (46.5)	

CVP: Central Venous Pressure; EF: Ejection Fraction; MCS: Mechanical Circulatory Support; mPAP: Mean Pulmonary Arterial Pressure; PADP: Pulmonary Arterial Diastolic Pressure; PAPS: Pulmonary Arterial Systolic Pressure; PCWP: pulmonary capillary wedge pressure; PVR: pulmonary vascular resistance; SD: standard deviation; TAPSE: Tricuspid Annular Plane Systolic Excursion; TAPSE/PASP: Tricuspid Annular Plane Systolic Excursion/ Pulmonary Artery Systolic Pressure

### Multivariable logistic regression

A multivariable logistic regression model was constructed to identify factors independently associated with intraoperative MCS use. Variables that reached statistical significance on univariate analysis (transplant laterality, preoperative PVR, LAS, and intraoperative mPAP) were entered into the model; intraoperative PASP and mPAP were collinear, and mPAP was retained as the more representative summary of intraoperative pulmonary arterial pressure. Adjusted odds ratios with 95% confidence intervals are presented in Table 5. After adjustment, higher preoperative PVR (adjusted OR 1.25 per 1 WU increase, 95% CI 1.03–1.52, p = 0.024), higher LAS (adjusted OR 1.06 per 1-point increase, 95% CI 1.04–1.08, p < 0.0001), and higher intraoperative mPAP (adjusted OR 1.1 per 1 mmHg increase, 95% CI 1.05–1.16, p < 0.0001) remained independently associated with intraoperative MCS use, while right-sided transplant showed a trend toward independent association (adjusted OR 2.26, 95% CI 0.996–5.107, p = 0.051).

**Table 5:** Multivariate Analysis: Independent Factors Associated with Intraoperative MCS Use During Single Lung Transplantation

Effect	Odds Ratio	95% CI	P-value
Right side transplant	2.26	0.996, 5.107	0.051
PVR	1.25	1.030, 1.525	0.024
LAS	1.06	1.037, 1.083	<0.0001
Intraoperative mPAP	1.10	1.050, 1.156	<0.0001

LAS: lung allocation score; mPAP: Mean Pulmonary arterial pressure; PVR: pulmonary vascular resistance

## Discussion

This study evaluating predictive use of preoperative hemodynamic and echocardiographic data to predict intraoperative MCS use in SLT demonstrated that a preoperative the RV-PA coupling ratio, or TAPSE/PASP <0.3 conferred a 4-fold increase in MCS use. In addition, invasive hemodynamics including PASP, mPAP, PADP, and PVR were all predictive of MCS use intraoperatively. Furthermore, while MCS use was associated with an increased hospital length of stay and longer duration of mechanical ventilation, there was no increase in short-term or long-term mortality.

MCS use has long been used to facilitate lung transplant in the setting of hypoxia or hemodynamic instability, initially by utilizing CPB and now more often via ECMO.^12^, ^13^, ^14^ The majority of the existing evidence on intraoperative MCS use and its indications has been derived from patients undergoing DLT with large primary PH population. The emergence of SLT as a viable alternative to DLT in patients with SPH necessitates an evaluation of current practice of MCS use intraoperatively, and identification of clinical variables that may be predictive of intraoperative MCS use within this patient population. Our center specifically, is a high-volume center, performing a large number of SLT due in part to our success with SLT as well as the advanced age often seen in our patients (mean age 66.2 ± 7.1).^1^, ^15^ In this study, we identified several factors that could predict MCS requirements *a priori* based on pre-operative and pre-incisional knowledge of the patient, in contrast with previously described mid-explant changes in intraoperative hemodynamics. The strongest predictors of intraoperative MCS use were invasive intra-operative pulmonary arterial hemodynamic measurements, LAS and, RV-PA coupling ratio. To the best of our knowledge, this study is the largest study in literature investigating MCS use in SLT procedures due to SPH.

### Hemodynamic and echocardiographic predictors of intraoperative MCS use in SLT

Prior studies have indicated that the severity of pulmonary hypertension with a mean PAP of 45 mmHg or greater correlates with the need for MCS during LT.^16^ Additionally, PASP and PVR have been shown to correlate with intraoperative MCS requirement.^16^, ^17^ In our study, pre-operative PCWP, pre-operative PVR (Table 2) and intraoperative PASP (Table 3) were independently associated with intraoperative MCS use. Prior work also suggested that intra-operative changes such as a decrease in cardiac index (CI) after PA clamping may be predictive of intraoperative MCS needs^18^ Shah et al reported pre-operative CI to be significantly lower in the ECMO group (3.2 vs 2.8, p<0.001), however this did not hold true for our study (2.4 vs 2.5, p = 0.1).^16^ This difference may be likely due to an overall lower preoperative CI amongst our cohort. Additionally, longstanding SPH could result in compensatory right ventricle hypertrophy (RVH) that would provide some protection in the setting of PA clamping to facilitate pneumonectomy.

Echocardiographic measurements assessing RV function have previously been examined as predictors for MCS use during lung transplantation with varied success and findings.^6^, ^16^ RV dilation, hypertrophy, degree of tricuspid regurgitation and global systolic function have been previously correlated with intraoperative MCS use during sequential DLT.^16^ While our work looked exclusively at SLT, similarities could exist since the initial pneumonectomy in a DLT may be analogous to a SLT. Prior work found that when looking at all-comers, most of whom received bilateral lung transplant, TAPSE was significantly different in the non-MCS and MCS groups (19.9 vs 17.8, p=0.04).^6^ In our study, variables derived from echocardiography alone, such as TAPSE and LVEF did not reach statistical significance in predicting intraoperative MCS use. When we dichotomized TAPSE into reduced and normal cohorts, MCS was utilized 20.9% for patients with reduced TAPSE and 12.6% for those with normal TAPSE (Table 4). One explanation as to why preoperative TAPSE in isolation did not predict MCS use in our study may be due to the ventricular remodeling and hypertrophy from longstanding PH, so that echocardiographic estimation of RV function using TAPSE alone may not accurately assess disease severity. Additionally, in long-standing PH, right ventricular remodeling and dysfunction may occur predominantly in the middle and apical portions of its free wall and be less pronounced at the base where TAPSE is measured.^19^ Finally, intraoperative changes after PA clamping and acute changes in RV afterload may be more reflective of its functional adaptability and help predict the need for MCS. This would limit the effectiveness of preoperative TAPSE as a surrogate for RV function during lung transplant. Other echocardiographic variables directly assessing RV such as fractional area change have not yet been fully studied as predictors of RV failure and subsequent hemodynamic instability requiring MCS for lung transplant but may be targets for future research.

RV – PA coupling ratio is derived from combining hemodynamic and echocardiographic information and helps assess RV-PA uncoupling. It has been used in various cardiothoracic and heart failure populations as an independent predictor of clinical outcomes.^20^, ^21^ Our study found that a TAPSE/PASP ratio <0.3 was significantly associated with increased intraoperative MCS requirements (OR 4.09 [95% 2.06–8.14]). This finding is of great significance as RV-PA uncoupling ratio, an easily measurable preoperative variable, can aid in defining selection standards and helps in establishing an evaluation benchmark. Previously, a TAPSE/PASP <0.3 has been shown to be associated with decreased transplant-free survival in patients awaiting lung transplantation.^22^, ^23^ Although prior work looked at transplant-free survival, it is quite notable that the same inflection point exists for intraoperative MCS use. This presents an opportunity for further investigations to examine this inflection point across the lung transplantation pathway.

#### Survival and outcomes analysis

The use of intraoperative MCS may add to the risks of vascular injury, thrombosis, intraoperative blood transfusions and bleeding, and systemic inflammation.^24^ While ECMO is associated with less inflammation than CPB, neither are harmless platforms.^12^, ^24^, ^25^, ^26^, ^27^ In our patients, the use of MCS was associated with a significantly longer hospitalization (MCS free: 19.1 [±18.5] days vs MCS assisted: 29.8 [±35.0] days p=0.007) as well as prolonged mechanical ventilation but not other comorbidities such as PGD Grade 3 requiring ECMO, tracheostomy or the need for hemodialysis. (Table 3). Additionally, survival out to 3 years was not significantly different (Figure 3). These findings are consistent with prior studies investigating both SLT and DLT populations as well as cannulation strategies.^10^, ^28^, ^29^ However, the Vienna group reported a survival benefit to intraoperative VA ECMO in an exclusively DLT population.^30^

The effects of MCS on graft function remains an area of ongoing investigation. The effects of MCS on ischemia/reperfusion injury remain unclear with reports of both increased and decreased rates after MCS use.^31^, ^32^ MCS has also been associated with an increased risk of severe PGD, although ECMO appears to confer less than CPB.^33^ Partly for this reason, recent ISHLT guidelines favor V-A ECMO over CPB during LT.^9^ In contrast, our data did not show any significant increase in severe PGD when using MCS (6.8% vs 4.4% without MCS p=0.49) Although we are unable to draw any significant conclusion regarding incidence of reperfusion injury from our work, one possible explanation for this difference is the Loor group looked at double lung transplants while our analysis was confined to SLT which are typically shorter in duration with shorter ischemic times. This is an open area for future work to investigate.

In our data, intraoperative MCS appears not to affect post-transplant survival, nor the need for postoperative tracheostomy or hemodialysis, and thus should be utilized judiciously in SLT procedures as indicated. The longer length of stay in MCS use patients might be related to higher rates of comorbidities in the cohort.

#### LAS and its components

The lung allocation score (LAS) representing the urgency of waiting list status and the likelihood of a candidate benefiting from transplant was significantly higher in the MCS group compared the non-MCS group (56.6 ± 20.4 vs 40.1 ±11.6, p<0.0001).^34^, ^35^ Additionally, our multivariate analysis showed that the use of intraoperative MCS increases by 1.06-point increase in LAS. Further, several individual components of LAS composite score, specifically PCWP, primary lung pathology as well as intraoperative PASP and mPAP were also independently predictive of MCS use. A higher LAS scores indicates more advanced lung disease, as indicated by it’s individual parameters, and thus necessitated more MCS use. Although LAS was replaced by CAS in March 2023, CAS has a significant overlap with LAS, thus our findings might be relevant with the new scoring system as well.^36^, ^37^, ^38^

### Limitations

This study has a large sample size; however, it is a single center study that may limit generalizability. Further, the study is limited by its retrospective nature and the implicit selection bias that all patients had to first qualify for transplant based on institutional criteria. Other transplant centers may have different screening thresholds for transplant candidacy that may entail different patient populations. The elapsed time between pre-operative TTE or RHC were not analyzed on a per-patient level so it is possible that some patients had changes from their pre-operative status that was not captured in our analysis. The decision to use MCS intraoperatively occurred at the joint discretion of the anesthesiologist and transplant surgeon, and there may be variability in thresholds for using MCS. Finally, to avoid a per-surgeon analysis, we grouped CPB and VA-ECMO into a single cohort, as there is surgeon-specific preference for one platform over another within our center.

## Conclusion

Assessment of preoperative RV-PA uncoupling can help predict intraoperative MCS use during SLT. A preoperative TAPSE/ PASP ratio <0.3 confers a 4-fold increase in the likelihood of MCS use. The use of MCS during SLT does increase length of stay, however, it does not significantly change survival, thus supporting its judicious use.

## Financial Disclosure Statement

No author reports any financial disclosures. No funding was obtained for this investigation.

## Declaration of Competing Interest

The authors declare that they have no known competing financial interests or personal relationships that could have appeared to influence the work reported in this paper.
